# Sexual activity and functioning in long-term breast cancer survivors; exploring associated factors in a nationwide survey

**DOI:** 10.1007/s10549-022-06544-0

**Published:** 2022-02-28

**Authors:** Solveig K. Smedsland, Kathrine F. Vandraas, Synne K. Bøhn, Alv A. Dahl, Cecilie E. Kiserud, Mette Brekke, Ragnhild S. Falk, Kristin V. Reinertsen

**Affiliations:** 1 National Advisory Unit of Late Effects after Cancer Treatment, Department of Oncology, Oslo University Hospital, Oslo, Norway; 2 General Practice Research Unit, Institute of Health and Society, University of Oslo, Oslo, Norway; 3 Research Support Services, Oslo Centre for Biostatistics and Epidemiology, Oslo University Hospital, Oslo, Norway

**Keywords:** Sexual function, Breast cancer survivorship, Late effects, Aromatase inhibitor

## Abstract

**Purpose:**

Sexual health is a key quality of life issue. Knowledge concerning sexual health in long-term breast cancer survivors (BCSs) is limited. Within a nationwide sample, we aimed to assess the prevalence of sexual inactivity and to explore factors associated with sexual inactivity and reduced sexual functioning among long-term BCSs.

**Methods:**

Long-term BCSs aged 20–65 years when diagnosed with early-stage breast cancer in 2011–2012 were identified by the Cancer Registry of Norway in 2019 (*n* = 2803) and invited to participate in a nationwide survey. Sexual health was measured using the multidimensional Sexual Activity Questionnaire. Factors associated with sexual inactivity and reduced sexual functioning were explored using multivariable logistic- and linear regression analyses with adjustments for relevant sociodemographic, health-, and cancer-related variables.

**Results:**

The final sample consisted of 1307 BCSs with a mean age of 52 years at diagnosis. Fifty-two percent of the BCSs were sexually inactive. Lack of interest was the most common reason for sexual inactivity. Treatment with aromatase inhibitor (OR 1.73, 95% CI 1.23, 2.43) and poor body image (OR 0.99, 95% CI 0.99, 0.995) were associated with sexual inactivity. Among sexually active BCSs, depression (B − 1.04, 95% CI − 2.10, − 0.02) and physical inactivity (B − 0.61, 95% CI − 1.21, − 0.02) were inversely related to sexual pleasure. Treatment with aromatase inhibitor (B 0.61, 95% CI 0.20, 1.01), sleep problems (B 0.37, 95% CI 0.04, 0.70), breast symptoms (B 0.01, 95% CI 0.003, 0.02), and chronic fatigue (B 0.43, 95% CI 0.05, 0.81) were associated with sexual discomfort. Chemotherapy (OR 1.91, 95% CI 1.23, 2.97), current endocrine treatment (OR 1.98, 95% CI 1.21, 3.25), and poor body image (OR 0.98, 95% CI 0.98, 0.99) were associated with less sexual activity at present compared to before breast cancer.

**Conclusion:**

Treatment with aromatase inhibitor seems to affect sexual health even beyond discontinuation. Several common late effects were associated with sexual inactivity and reduced sexual functioning. To identify BCSs at risk of sexual dysfunction, special attention should be paid to patients treated with aromatase inhibitor or suffering from these late effects.

## Introduction

Due to advances in diagnostics and treatment, the five-year relative survival rate for early-stage breast cancer (BC) has surpassed 90% in the Western world [1, 2]. The number of long-term breast cancer survivors (BCSs) (i.e., more than five years since diagnosis) is steadily increasing, and research concerning different aspects of survivorship care is of considerable interest.

Sexual health, defined as a state of physical, emotional, mental, and social well-being in relation to sexuality [3], is an important aspect of quality of life [4, 5]. Female sexual dysfunction includes lack of sexual interest and arousal, inability to achieve orgasm, and pain during intercourse [6]. Reasons for sexual dysfunction are multifactorial, including biological, psychological, interpersonal, and sociocultural factors [7].

In the general female population the prevalence of sexual dysfunction is estimated at 40–50% based on a consensus statement [8]. BCSs face challenges related to BC treatment and to late effects of different treatment modalities that may further negatively affect their sexual health. BC treatment is often intensive, including combinations of surgery, radiotherapy, and systemic therapies. Surgery and radiotherapy may result in physical changes such as loss of erogenous zones or scarring and in psychological challenges, such as altered body image [9, 10]. Chemotherapy-induced premature menopause or estrogen deprivation therapy may affect sexual health both directly through the effects on genital tissues and indirectly as troublesome vasomotor symptoms and sleep problems [11]. In the post-treatment phase, many BCSs struggle with late effects, such as chronic fatigue (CF) and persistent mental distress [12], which also may affect their sexual health in a negative way. Combined, BCSs represent a particularly vulnerable group with regards to impaired sexual health.

Sexual dysfunction is frequently reported among BCSs with prevalence of 73% in a recent meta-analysis [13]. Prevalence estimates differ, however, widely across studies from 27% [14] to 93% [15], primarily reflecting methodological differences. Most studies focus on sexual health during the first few years after BC diagnosis [15–19] and therefore research-based knowledge concerning sexual health among long-term BCSs is limited [20–24]. Furthermore, how different BC treatments (surgery, chemotherapy, and endocrine therapy) contribute to sexual dysfunction at long term is still unclear [5, 24–28].

In order to improve the quality of survivorship care in long-term BCSs, these knowledge gaps need to be addressed. An important step in that direction is to identify factors associated with poor sexual health, as such information may aid clinicians dealing with this growing survivor population.

The aim of this study was twofold; firstly, to describe different aspects of sexual health in a nationwide sample of long-term BCSs by assessing the prevalence and reasons for sexual inactivity and secondly, to explore factors associated with sexual inactivity and reduced sexual functioning.

## Materials and methods

### Study population

This study is part of the SWEET study (**s**urvivorship **w**ork-s**e**xual h**e**al**t**h-study), a cross-sectional questionnaire study examining work life and sexual health among Norwegian long-term BCSs. All women diagnosed with BC stage I–III in 2011 or 2012 at the age of 20–65 years were identified by the Cancer Registry of Norway (CRN). CRN is based on mandatory reporting and has, as from when it was established in 1951, close to complete registration of all new cancer cases in Norway [29]. To be included in the study, women had to be free of pre- or post-malignancies (except non-melanoma skin cancer and ductal carcinoma in situ). Invitation was mailed to 2803 BCSs during December 2019. One reminder was sent to non-responders (*n* = 1684) in February 2020.

### Primary outcomes

The Sexual Activity Questionnaire (SAQ) [30] was used to assess the prevalence of sexual inactivity, reasons for sexual inactivity, and different aspects of sexual functioning among the sexually active BCSs. The SAQ is reported to have good psychometric properties in the general population [31] and has been used in several BC-specific settings [5, 20, 23, 32].

The first part of the SAQ assesses whether women are sexually active. Sexually active is defined as being sexually engaged with a partner. In the second part eight reasons for eventual sexual inactivity are listed, and the sexually inactive women tick the reasons that apply to them. The third part measures sexual functioning (SAQ-F) during the last month among sexually active women across four subscales: pleasure (SAQ-P), discomfort (SAQ-D), habit (SAQ-H), and tiredness. SAQ-H was modified from “How did the frequency of sexual activity compare with what is usual for you?” to “How often are you engaged in sexual activity compared to before the BC diagnosis?” Responses to the SAQ-F items are scored on a 4-point scale ranging from 0 to 3 and summarized within each subscale. A higher sum score indicates greater pleasure, more discomfort, more sexual activity, and more tiredness. The SAQ-P consists of six items with sum score ranging from 0 to 18. The SAQ-D consists of two items with sum score ranging from 0 to 6. The SAQ-H and tiredness-scale consist of one item each with sum score from 0 to 3. Cronbach’s alpha for SAQ-F was 0.81.

### Explanatory variables

Socio-demographic information was self-reported and included age at survey, living with a partner or not, living with children < 18 years or not, educational level (≤ 12 years/ > 12 years), and paid work status (full-time work, part-time work, self-employment, and workers on sick leave) versus not (disability pension, retirement) at survey.

Cancer-related variables (BC stage, hormone receptor-, and human epidermal growth factor receptor 2 (HER2) status), age at diagnosis, and type of surgery were obtained from the CRN. Information on chemotherapy, radiation therapy, and endocrine therapy (tamoxifen, aromatase inhibitor (AI)) was based on self-report.

The presence of self-reported somatic comorbidity included 17 questions on major somatic conditions (cardiovascular, pulmonary, thyroid, kidney, gastro-intestinal-, or rheumatic disease, diabetes, arthrosis, muscle/joint pain, and epilepsy). Affirmative responses were categorized into no comorbid condition, 1–2 or ≥ 3 comorbid conditions.

Sleep problems were defined as more than three episodes per week of difficulty falling asleep and/or waking up too early without going back to sleep for the past three months [33].

Pain was assessed using the European Organization for Research and Treatment of Cancer Quality of Life Questionnaire (EORTC-QLQ C30 version 3) [34], while breast symptoms and body image (BI) were assessed by the EORTC-QLQ breast cancer-specific module—BR23 [35]. Items are rated from 1 (not at all) to 4 (very much) and then transformed to 0–100 scales according to manuals. Higher scores correspond to more pain, more breast symptoms, and better BI.

The Fatigue Questionnaire (FQ) [36] measures fatigue symptoms during the past month through eleven items; seven on physical and four on mental fatigue. Responses are rated from 0 (less than usual) to 3 (much more than usual) and summarized, yielding sum scores from 0 to 33. A higher score indicates more fatigue. Cases with CF were identified by a dichotomized score for each response alternative, resulting in sum scores from 0 to 11. CF was defined as a sum score ≥ 4 with duration six months or more [37]. Cronbach’s alpha was 0.93 for total fatigue.

Height and weight were self-reported. Obesity was defined as body mass index (BMI) ≥ 30 kg/m^2^ [38].

“Physically inactive” was defined as not meeting the public guidelines of ≥ 150 min moderate-intensity physical activity or ≥ 75 min of high-intensity physical activity per week or an equivalent combination of moderate- and high-intensity physical activity per week [39], using a modified version of the Godin Leisure Time Questionnaire [40].

Anxiety was assessed by the General Anxiety Disorder 7-item scale (GAD-7) covering the last two weeks. All items are rated from 0 (not at all) to 3 (nearly every day) resulting in sum scores from 0 to 21. The presence of generalized anxiety disorder was defined as a sum score ≥ 10 [41]. Cronbach’s alpha was 0.87.

Depression was measured by The Patient Health Questionnaire-9 (PHQ-9) assessing symptom severity during the past two weeks by nine items rated from 0 (not at all) to 3 (nearly every day) resulting in sum scores from 0 to 27. Major depressive episode was defined as a sum score ≥ 10 [42]. Cronbach’s alpha was 0.85.

### Statistical analysis

Missing data were handled according to the respective manuals. When at least 50% of the items had been completed, mean imputation procedures were performed for the EORTC-QLQ C 30 and BR 23, the GAD-7, and within each subscale for the FQ. For the PHQ-9 mean imputation procedure was performed if no more than two items were missing. For the subscales of the SAQ-F, responders with missing items were excluded from the analyses.

Descriptive statistics are presented as frequencies and proportions for categorical data, and as mean and standard deviation (SD) for continuous data. Comparisons of sexually active and inactive BCSs were performed by independent sample t-tests and chi square tests as appropriate.

Factors associated with sexual inactivity were identified using logistic regression analyses, while factors associated with SAQ-P, SAQ-D, and tiredness were identified using linear regression analyses.

Due to a highly skewed distribution of SAQ-H, this variable was dichotomized into “less sexual activity now compared to before BC” versus “same/some more/much more sexual activity compared to before BC” and analyzed using logistic regression analyses.

Both univariate and multivariable regression analyses were performed. Variables with *p* value < 0.20 in the univariate analyses were included in the multivariable models. Age at diagnosis and living with children under 18 years were omitted due to high correlations with age at survey, but otherwise no multicollinearity was observed. Due to the large sample size, no backward elimination was performed to avoid exclusion of important factors associated with sexual health. The assumption of linearity was fulfilled for all the continuous variables.

Results were presented as beta coefficients (B) for linear regression and odds ratio (OR) for logistic regression analyses with accompanying 95% confidence intervals (CI). *p* values < 0.05 were considered statistically significant.

To explore potential selection bias of our sample, we compared registry information of responders versus non-responders.

All analyses were performed using IBM SPSS statistics version 26.0 (Armonk, NY).

## Results

### Patient characteristics

Of the 2803 BCSs invited, 1361 returned the questionnaire (49%). We excluded six BCSs with either incomplete consent or self-reported BC recurrence, in addition to 48 BCSs with incomplete information on sexual activity, resulting in a final sample of 1307 women.

Mean age at diagnosis was 51.7 (SD 8.6) years and 59.7 (SD 8.7) years at survey. Most participants lived with a partner (74%) and had been treated for BC stage I or II (81%) with breast-conserving therapy (59%), radiotherapy (80%), endocrine therapy (65%), and chemotherapy (69%). Twenty-three percent reported current use of endocrine therapy (Table 1).

**Table 1:** Characteristics of the total sample and the subgroups of sexually active and inactive breast cancer survivors

Variables	Total sample *n* = 1307	Sexually active *n* = 627	Sexually inactive *n* = 680	*p* value
Socio-demographic variables				
Age at diagnosis (years), mean (SD)	51.7 (8.6)	50.0 (8.7)	53.3 (8.2)	** < 0.001**
Age at survey (years), mean (SD)	59.7 (8.7)	58.0 (8.8)	61.3 (8.3)	** < 0.001**
Living with spouse/partner, *n* (%)	966 (74)	555 (89)	411 (60)	** < 0.001**
Living with children < 18 years, *n* (%)	199 (15)	118 (19)	81 (12)	**0.001**
Education > 12 years, *n* (%)	671 (52)	354 (57)	317 (47)	**0.001**
Paid work at survey, *n* (%)	545 (43)	308 (50)	237 (36)	** < 0.001**
Cancer-related variables				
Stage^a^				0.92
I, *n* (%)	583 (45)	282 (45)	301 (44)	
II, *n* (%)	470 (36)	228 (36)	242 (36)	
III, *n* (%)	105 (8)	49 (8)	56 (8)	
*Missing*	*149*	*68*	*81*	
Hormone receptor positive, *n* (%)	1111 (85)	523 (84)	588 (87)	0.12
HER-2^b^ positive, *n* (%)	241 (18)	117 (19)	124 (18)	0.98
Triple negative, *n* (%)	112 (9)	60 (10)	52 (8)	0.22
Surgery				0.62
Mastectomy, *n* (%)	537 (41)	262 (42)	275 (40)	
Breast-conserving therapy, *n* (%)	770 (59)	365 (58)	405 (60)	
Chemotherapy, *n* (%)	895 (69)	429 (68)	466 (69)	0.97
Radiotherapy, *n* (%)	1047 (80)	504 (80)	543 (80)	0.81
Endocrine treatment (ET)				** < 0.001**
No ET, *n* (%)	456 (35)	227 (36)	229 (34)	
Aromatase inhibitor, *n* (%)	404 (31)	159 (25)	245 (36)	
Tamoxifen, *n* (%)	378 (29)	214 (34)	164 (24)	
Unknown type, *n* (%)	69 (5)	27 (4)	42(6)	
ET at present, *n* (%)	295 (23)	157 (25)	138 (20)	**0.04**
Health variables				
Somatic comorbidity				** < 0.001**
No condition, *n* (%)	281 (22)	166 (27)	115 (17)	
1–2 condition(s), *n* (%)	706 (54)	344 (55)	362 (54)	
≥ 3 conditions, *n* (%)	313 (24)	114 (18)	199 (29)	
*Missing*	*7*	*3*	*4*	
Sleep problems, *n* (%)	571 (44)	243 (39)	328 (49)	** < 0.001**
Pain^c^, mean (SD)	28.0 (29.3)	24.4 (27.8)	31.4 (30.2)	** < 0.001**
Breast symptoms^c^, mean (SD)	16.0 (19.0)	14.3 (17.6)	17.5 (20.0)	**0.002**
Body image^c^, mean (SD)	75.8 (26.2)	79.0 (24.7)	72.9 (27.2)	** < 0.001**
Chronic fatigue, *n* (%)	420 (33)	177 (29)	243 (37)	**0.002**
Obesity^d^, *n* (%)	234 (18)	91 (15)	143 (22)	**0.001**
Physically inactive, *n* (%)	693 (53)	307 (49)	386 (57)	** < 0.001**
Anxiety disorder, *n* (%)	94 (7)	35 (6)	59 (9)	**0.03**
Major depression, *n* (%)	238 (19)	87 (14)	151 (23)	** < 0.001**

*Bold* statistically significant (*p* < 0.05)

*SD* standard deviation

^a^Based on TNM

^b^HER-2 = human epidermal growth factor receptor 2

^c^Scale 0–100 (a higher score corresponds to more pain and breast symptoms and a better body image)

^d^Body mass index ≥ 30 kg/m^2^

### The sexually inactive BCSs

About half (52%) of the BCSs were sexually inactive. Prevalence rate was highest among the oldest BCSs, ranging from 32% among those aged 30–39 years, 56% among those aged 60–69 years, and 67% in the oldest age group (70–74 years). Older age (OR 1.05, 95% CI 1.02, 1.07), living without a partner (OR 5.19, 95% CI 3.75, 7.19), and treatment with AI (OR 1.73, 95% CI 1.23, 2.43) were positively associated with sexual inactivity in multivariable analyses. Better BI was negatively associated with sexual inactivity (OR 0.99, 95% CI 0.99, 0.995) (Table 2).

**Table 2:** Factors associated with sexual inactivity in breast cancer survivors (sexual activity as reference)

Variables	Bivariate analysis		Multivariable analysis		
	OR	95% CI	OR	95% CI	*p* value
Age at survey (years)	**1.05**	**1.03, 1.06**	**1.05**	**1.02, 1.07**	** < 0.001**
Not living with partner	**5.05**	**3.78, 6.74**	**5.19**	**3.75, 7.19**	** < 0.001**
Education ≤ 12 years	**1.47**	**1.18, 1.83**	1.08	0.83, 1.41	0.56
No paid work at survey	**1.81**	**1.45, 2.27**	0.93	0.68, 1.26	0.62
Mastectomy (BCT = ref)	0.95	0.76, 1.18	–	–	–
Chemotherapy	1.01	0.80, 1.27	–	–	–
Radiotherapy	0.97	0.74, 1.27	–	–	–
Endocrine treatment (ET)					
No ET (ref)	–	–	–	–	–
Aromatase inhibitor	**1.53**	**1.17, 2.00**	**1.73**	**1.23, 2.43**	**0.002**
Tamoxifen	0.76	0.58, 1.00	1.03	0.71, 1.51	0.87
Unknown type	1.54	0.92, 2.59	1.24	0.66, 2.33	0.50
ET at present	**0.76**	**0.59, 0.99**	1.09	0.76, 1.56	0.64
Somatic comorbidity					
No disease (ref)	–	–	–	–	–
1–2 comorbid disease(s)	**1.52**	**1.15, 2.01**	1.22	0.88, 1.71	0.24
≥ 3 comorbid diseases	**2.52**	**1.81, 3.51**	1.29	0.83, 2.00	0.26
Sleep problems	**1.49**	**1.20, 1.86**	1.15	0.87, 1.52	0.32
Pain^a^	**1.01**	**1.01, 1.01**	1.00	1.00, 1.01	0.74
Breast symptoms^a^	**1.01**	**1.00, 1.02**	1.00	1.00, 1.01	0.46
Body image^a^	**0.99**	**0.99, 0.995**	**0.99**	**0.99, 0.995**	**0.003**
Chronic fatigue	**1.44**	**1.14, 1.82**	1.30	0.95, 1.79	0.10
Obesity	**1.60**	**1.20, 2.13**	1.30	0.91, 1.85	0.15
Physically inactive	**1.58**	**1.26, 1.98**	1.21	0.93, 1.58	0.16
Anxiety disorder	**1.62**	**1.05, 2.50**	1.13	0.65, 1.99	0.66
Major depression	**1.82**	**1.36, 2.43**	1.20	0.79, 1.83	0.39

*Bold* statistically significant (*p* < 0.05)

*OR* odds ratio, *CI* confidence interval, *BCT* breast-conserving therapy

^a^Scale 0–100 (a higher score corresponds to more pain and breast symptoms and a better body image).

**Fig. 1:**
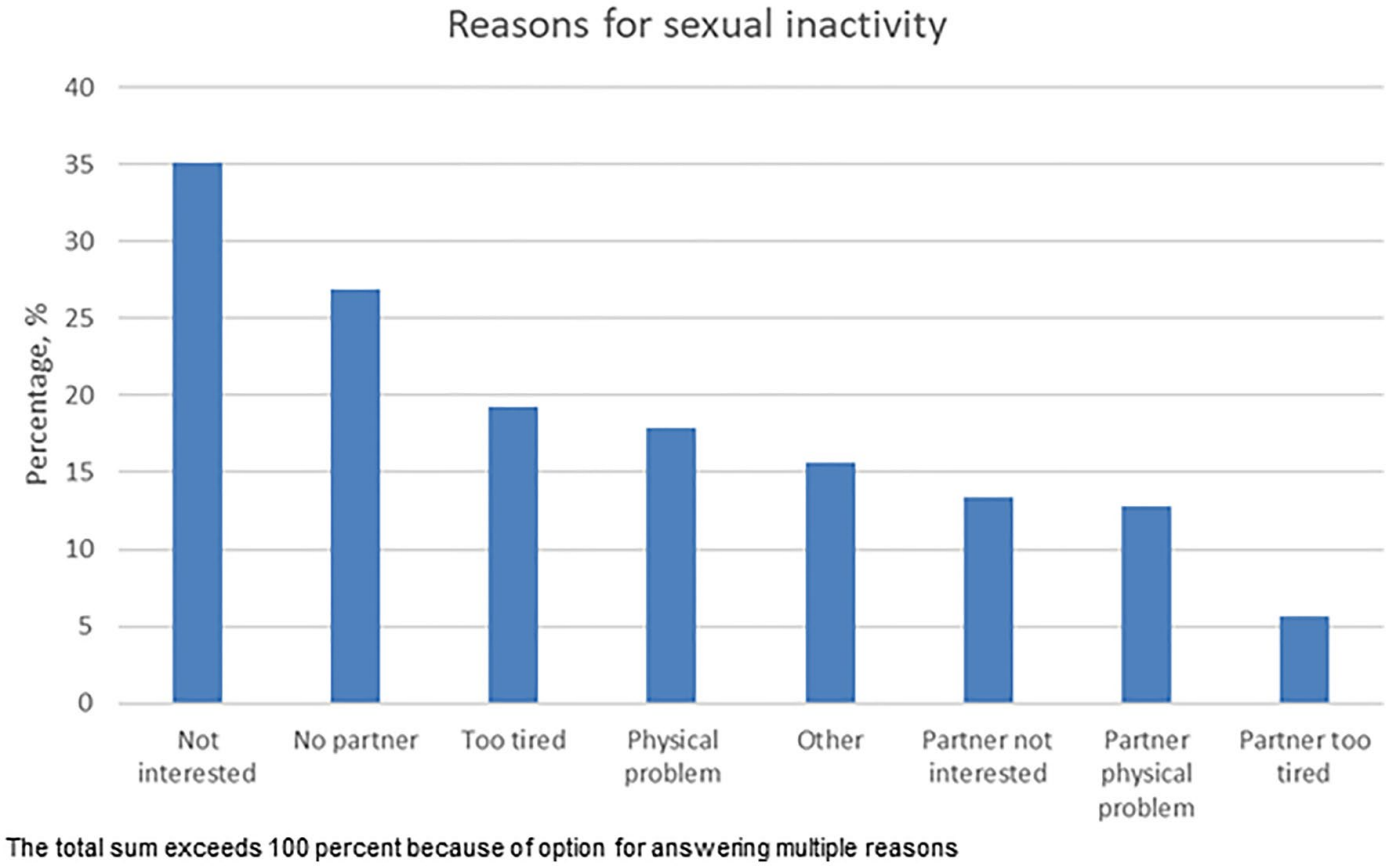
Reasons for being sexually inactive in sexually inactive breast cancer survivors

The most common reasons for sexual inactivity were lack of interest (35%), lack of partner (27%), being too tired (19%), and having a physical problem (18%). Partner issues were reported by 25% (Fig. 1).

### Sexual functioning among the sexually active BCSs (i.e., sexually engaged with a partner)

Among sexually active BCSs, 555 (89%) lived with a partner, while 72(11%) did not.

Mean SAQ-P score was 10.8 (SD = 3.7). Living without a partner was positively associated with sexual pleasure (B 1.68, 95% CI 0.78, 2.58), while physical inactivity (B − 0.61, 95% CI − 1.21, − 0.02) and depression (B − 1.04, 95% CI − 2.10, − 0.02) were negatively associated with sexual pleasure in multivariable analyses (Table 3).

**Table 3:** Factors associated with sexual functioning (SAQ-F) subscales in sexually active breast cancer survivors

Variables	Pleasure (SAQ-P)^a^ Linear regression		Discomfort (SAQ-D)^b^ Linear regression		Tiredness^c^ Linear regression		Habit (SAQ-H)^d,e^ Logistic regression	
	B	95% CI	B	95% CI	B	95% CI	OR	95% CI
Age at survey (years)	–	–	–	–	**− 0.02**	**− 0.03, − 0.004**	1.00	0.97, 1.03
Not living with partner	**1.68**	**0.78, 2.58**	**− 0.87**	**− 1.33, − 0.41**	**− 0.38**	**− 0.59, − 0.16**	**0.40**	**0.23, 0.69**
Education ≤ 12 years	–	–	–	–	–	–	–	–
No paid work at survey	–	–	–	–	–	–	–	–
Mastectomy (BCT = ref)	− 0.04	− 0.78, 0.71	0.20	− 1.18, 0.59	0.04	− 0.14, 0.22	1.10	0.74, 1.62
Chemotherapy	− 0.33	− 1.05, 0.40	0.19	− 0.18, 0.57	0.13	− 0.05, 0.31	**1.91**	**1.23, 2.97**
Radiotherapy	0.69	− 0.18, 1.55	− 0.28	− 0.72, 0.17	− 0.05	− 0.25, 0.15	–	–
Endocrine treatment (ET)								
No ET (ref)	–		–		–		–	
Aromatase inhibitor	− 0.63	− 1.42, 0.16	**0.61**	**0.20, 1.01**	0.11	− 0.08, 0.31	1.01	0.62, 1.64
Tamoxifen	0.06	− 0.78, 0.89	0.07	− 0.36, 0.50	0.05	− 0.15, 0.24	0.65	0.40, 1.08
Unknown type	0.74	− 0.76, 2.23	− 0.30	− 1.08, 0.48	− 0.04	− 0.40, 0.31	0.96	0.39, 2.37
ET at present	− 0.38	− 1.19, 0.43	0.28	− 0.13, 0.69	− 0.01	− 0.19, 0.18	**1.98**	**1.21, 3.25**
Somatic comorbidity								
No disease (ref)	–	–	–		–		–	
1–2 comorbid disease(s)	–	–	0.08	− 0.29, 0.45	0.08	− 0.09, 0.24	0.85	0.56, 1.29
≥ 3 comorbid diseases	–	–	0.35	− 0.18, 0.88	0.01	− 0.23, 0.25	1.49	0.80, 2.78
Sleep problems	− 0.33	− 0.97, 0.32	**0.37**	**0.04, 0.70**	0.04	− 0.12, 0.19	1.02	0.69, 1.50
Pain^f^	0.003	− 0.01, 0.02	0.003	− 0.004, 0.01	0.003	0.000, 0.006	1.00	0.99, 1.01
Breast symptoms^f^	–	–	**0.01**	**0.003, 0.02**	− 0.001	− 0.006, 0.003	1.00	0.99, 1.01
Body image^f^	0.001	− 0.01, 0.02	− 0.01	− 0.01, 0.003	− 0.002	− 0.005, 0.001	**0.98**	**0.98, 0.99**
Chronic fatigue	− 0.56	− 1.30, 0.17	**0.43**	**0.05, 0.81**	**0.33**	**0.16, 0.50**	1.54	0.99, 2.40
Obesity	–	–	**− 0.63**	**− 1.07, − 0.19**	–	–	–	–
Physically inactive	**− 0.61**	**− 1.21, − 0.02**	–	–	–	–	–	–
Anxiety disorder	− 0.34	− 1.70, 1.03	0.26	− 0.46, 0.97	0.18	− 0.14, 0.51	0.87	0.36, 2.12
Major depression	**− 1.04**	**− 2.10, − 0.02**	− 0.44	− 0.97, 0.09	0.21	− 0.03, 0.45	1.01	0.53, 1.93

Results from multivariable analyses adjusted for all variables listed. Statistically significant (*p* < 0.05) associations are indicated in bold

*B* beta coefficient,* CI* confidence interval, *OR* odds ratio, *BCT* breast-conserving therapy

^a^Adjusted *R*^2^ 0.059

^b^Adjusted *R*^2^ 0.125

^c^Adjusted *R*^2^ 0.158

^d^Nagelkerke R^2^ 0.174

^e^SAQ-H dichotomized into less vs same/more (reference) sexual activity after breast cancer diagnosis

^f^Scale 0–100 (a higher score corresponds to more pain and breast symptoms and a better body image)

Mean SAQ-D score was 2.2 (SD = 1.9). Treatment with AI (B 0.61, 95% CI 0.20, 1.01), sleep problems (B 0.37, 95% CI 0.04, 0.70), breast symptoms (B 0.01, 95% CI 0.003, 0.02), and CF (B 0.43, 95% CI 0.05, 0.81) were positively associated with sexual discomfort, while living without a partner (B − 0.87, 95% CI − 1.33, − 0.41) and obesity (B − 0.63, 95% CI − 1.07, − 0.19) were negatively associated with discomfort in multivariable analyses (Table 3).

Mean tiredness score was 1.2 (SD = 0.9). CF (B 0.33, 95% CI 0.16, 0.50) was positively associated with tiredness related to sex, while older age (B − 0.02, 95% CI − 0.03, − 0.004) and living without a partner (B − 0.38, 95% CI − 0.59, − 0.16) were negatively associated with tiredness in multivariable analyses (Table 3).

Fifty-four percent of the sexually active BCSs reported lower frequency of sexual activity at survey compared to before BC. Chemotherapy (OR 1.91, 95% CI 1.23, 2.97) and current endocrine therapy (OR 1.98, 95% CI 1.21, 3.25) were positively associated with less sexual activity, while living without a partner (OR 0.40, 95% CI 0.23, 0.69) and a better BI (OR 0.98, 95% CI 0.98, 0.99) were negatively associated with less sexual activity after BC in multivariable analyses (Table 3).

As shown in Table 3, explained variance in the models varied from 0.059 to 0.158.

### Attrition analysis

Information about non-responders (*n* = 1448) was limited to cancer-related information obtained from the CRN. Responders yielded similar results as non-responders for all variables except for age at diagnosis (51.7 years versus 53.2 years, *p* < 0.001), HER2 positivity (20% versus 15%, *p* < 0.001), and mean value of the proliferation marker Ki67 (31 versus 27, *p* < 0.001).

## Discussion

Approximately half of the BCSs were sexually inactive eight years after diagnosis, with highest prevalence among the oldest. Lack of interest was the most common reason for sexual inactivity. AI therapy was the most important treatment modality negatively affecting sexual health. Several individual and potential modifiable factors such as a poor BI, CF, depression, sleep problems, breast symptoms, and physical inactivity were associated with different aspects of sexual functioning.

As stated, studies concerning sexual activity and functioning in long-term BCSs are few. Only two other studies report prevalence rates of sexual inactivity and these rates are in line with our findings [21, 23]. Lack of interest was the most common reported reason for sexual inactivity both in our study and in another study using the SAQ among BCSs three years after diagnosis [5].

Reported prevalence rates of sexual inactivity in the general population are higher in older than younger age groups [43]. Normative data for the SAQ from a random sample of Norwegian women showed that 52% in the age group 56–69 years were sexually inactive [31]. In our study, the prevalence rate of sexually inactive BCSs in this age group was quite similar (54%). However, among those aged 35–44 years, the proportion of sexually inactive women was considerably higher among the BCSs in our study (33%) compared to the normative sample (16%). This finding is supported by another study of long-term BCSs where pre/peri-menopausal BCSs were less likely to be sexually active compared to corresponding controls, while no significant difference in sexual activity was observed between the post-menopausal groups [23]. Reasons for sexual inactivity were different in the Norwegian normative sample compared to in our study. In the normative sample the most common reason for sexual inactivity was lack of partner (48%), while only 19% reported that sexual inactivity was due to lack of interest [31].

AI therapy was associated with both sexual inactivity and more sexual discomfort in our study. Vaginal dryness, dyspareunia, and reduced libido are common adverse effects during AI treatment [44, 45] and in the first years after discontinuation [46]. Knowledge concerning sexual activity and functioning in long-term BCSs after discontinuation of adjuvant AI is missing. In our study, 78% of the BCSs treated with AI had discontinued the treatment. Thus our results are relevant for what happens after the adjuvant treatment period. Soldera et al., exploring sexual health in BCSs 12.5 years after diagnosis, found no significant differences in sexual activity according to former receipt of adjuvant endocrine treatment [23]. In that study the participants had used tamoxifen, which to a lesser extent cause vaginal dryness and dyspareunia compared to AI [44]. Davis et al. compared post-menopausal symptoms in long-term BCSs with controls and found worse sexual functioning in BCSs [22]. As BCSs treated with chemotherapy and still on endocrine treatment were excluded in that sub-analysis, the authors concluded that severe menopausal symptoms may persist even after cessation of endocrine treatment. Our findings support this viewpoint.

In our study, chemotherapy and current use of endocrine therapy were associated with less sexual activity eight years after diagnosis compared to before BC diagnosis. A larger proportion of BCSs < 55 years compared to BCSs ≥ 55 years at diagnosis received adjuvant chemotherapy in the present study, indicating that chemotherapy-induced premature menopause may be a possible explanation. BCSs still on adjuvant endocrine treatment are younger, adding to our findings that the youngest BCSs are especially vulnerable to sexual challenges after BC.

A poorer BI was associated with sexual inactivity and reduced sexual activity compared to before BC, which is well known from previous studies [18, 47, 48]. Depression was associated with lower sexual pleasure. In the general population there is a known bidirectional relation between depression and sexual dysfunction [49] and former studies of BCSs have showed an association with depression and lower sexual interest and desire [25, 32]. As expected, CF was associated with sexual tiredness. CF was also associated with more sexual discomfort, as were sleep problems and breast symptoms. We have found only one prior study reporting a relation between sexual dysfunction and fatigue in BCSs, and this study examined young BCSs one year after diagnosis [17]. Another study examining BCSs on average three years after diagnosis showed a relation between sleep problems and sexual discomfort, but not between fatigue and sexual functioning [50].

Physical inactivity was associated with lower sexual pleasure. A recent review stated that physical activity improves menopausal symptoms in the general female population, and indirectly physical activity may improve sexual functioning [51]. Results from a pilot study randomizing BCSs with menopausal symptoms to a lifestyle program including physical activity or standard care support this statement, demonstrating clinically significant reduction in both menopausal symptoms and sexual dysfunction in the intervention group [52]. Another study, exploring the associations of BMI, physical activity, and sexual dysfunction in BCSs, found that regular physical activity was associated with better sexual desire [53]. Further, physical activity may alleviate symptoms of depression [54], fatigue [55], and sleep problems [56], with the potential of indirectly improving sexual functioning.

Obesity was somewhat surprisingly associated with less sexual discomfort. This relation has been shown in one former study of BCSs [57]. Theoretically this finding may be explained by increased levels of circulating estrogen due to excessive aromatization activity in the adipose tissue [58]. Even though this theory was not verified in the above-mentioned study [57], it will be interesting to explore further in upcoming studies.

Living without a partner was as expected associated with sexual inactivity. More surprisingly, the sexually active women not living with a partner reported better sexual functioning across all domains than those living partnered. Similar associations have been shown in a study of BCSs four years after diagnosis. In that study, BCSs who had a partner they did not live with, had fewer problems related to desire, excitement, and lubrication compared to those living with a partner [26]. Further, a study of midlife women in the general population found that being partnered was associated with hypoactive sexual desire dysfunction.[59]. As only 72 of the sexually active BCSs in our study lived without a partner, these findings were not further explored.

### Strengths and limitations

This study is based on a nationwide sample of all Norwegian BCSs diagnosed in 2011 or 2012 registered in the CRN. The response rate was 49%, which is considered acceptable and comparable to other large-scale surveys on long-term survivors in Norway [60, 61] and cross-sectional studies on sexual health [5, 28]. Questionnaires with established psychometric properties were used. This is the first study to explore sexual health in long-term BCSs treated with modern BC therapies separating between different endocrine treatments. Few other studies have included both sociodemographic, treatment related, somatic-, and mental health-related variables to explore sexual outcomes in this population.

There are several limitations that need to be considered. Cross-sectional design precludes conclusions on causality. Furthermore, the study did not include a control group. Some findings are compared to normative Norwegian data, but comparisons with a matched control group from same time period would have strengthened the study. Lack of information on menopausal status is a limitation, as menopausal status obviously affect sexual functioning. On the other hand, adjustments for menopausal status may have disguised the effect of chemotherapy-induced menopause and endocrine deprivation therapy in premenopausal BCSs. Sexual activity and function were measured with the SAQ which has a rather narrow definition of sexually activity restricted to partnered sex and do not capture all elements of sexual activities. We cannot rule out that selection bias exists as we only had access to cancer-related variables for the non-responders. Given that a larger proportion of responders were HER2 positive and the mean Ki67 was higher, a higher proportion of responders may have received chemotherapy. Many variables in this study were based on patient-reported outcome measures, with the inherent risk of recall bias. Additionally, questionnaires concerning sexual health issues have a special risk of reporting bias [62]. BCSs > 65 years at diagnosis and BCSs with relapse or metastatic disease were not invited in the study. Thus, the results cannot automatically be generalized to the oldest BCSs or to BCSs with advanced disease.

Regression analyses showed a lower degree of explained variance for sexual pleasure compared to the other domains of sexual functioning. This could partly be due to the simplified model (where all variables were assumed to be independent), but could also indicate that other factors not included in the model may be important. Unfortunately, we had no information about the length, quality, and satisfaction of couple relationships or any sexual problems experienced by the partner, which are important predictors of sexual health in BCSs [14, 25, 32, 50, 63].

## Conclusion

Addressing sexual health issues should be a part of the standard follow-up of BCSs, even several years after treatment cessation. Specific attention should be paid to younger BCSs and those treated with AI. BCSs with gynecological symptoms should be offered treatment, and if using adjuvant AI, a switch to tamoxifen may be discussed. A poor body image, physical inactivity, depression, sleep problems, breast symptoms, and chronic fatigue should be assessed and handled as factors that may improve sexual health.

## Data Availability

All data are available at the National Advisory Unit for Late Effects after Cancer Treatment, Department of Oncology, Oslo University Hospital, the Radium Hospital, Oslo, Norway.
